# Deep learning and firearm wound classification: a pilot study

**DOI:** 10.3389/fmed.2025.1646656

**Published:** 2026-02-02

**Authors:** Giuseppe Delogu, Nicola Di Fazio, Gabriele Licciardello, Paola Frati, Cristoforo Pomara, Francesco Sessa

**Affiliations:** 1 Department of Anatomical, Histological, Forensic and Orthopedic Science, Sapienza University, Rome, Italy; 2 Department of Life Sciences, Health and Health Professions, Link Campus University, Rome, Italy; 3 Department of Medical, Surgical and Advanced Technologies “G.F. Ingrassia”, University of Catania, Catania, Italy

**Keywords:** artificial intelligence, deep learning, diagnosis, firearm, forensics, gunshot wound, pathology, pattern recognition

## Abstract

**Introduction:**

The study on the use of deep learning in pattern recognition of gunshot wounds (GSW) represents a novelty in the field of forensic pathology. Although artificial intelligence (AI) has already revolutionized many medical specialties, applications in forensic medicine are still limited. Nevertheless, AI-based tools could be of great use in a discipline that relies heavily on visual analysis. Scant scientific evidence from recent experimental studies has demonstrated the AI potential in predicting shooting distance based on GSW pictures. Previous approaches achieved a classification accuracy of 98%; however, further studies are needed to evaluate its applicability in forensic practice. The aim of this project is to further explore the application of deep learning techniques for the classification of GSWs.

**Materials and methods:**

The study, conducted at the University of Catania, employed the free software Lobe AI. It was necessary to proceed through 4 consecutive phases: training, model validation, testing, and data analysis. Four study categories were then identified: GSWs, entrance/exit wounds, wounds based on the pathological range of fire, and wounds based on the type of weapon ammunition. For the software training, educational images were extracted from a forensic atlas specializing in GSWs. For the testing phase, photos from the Catania forensic case history and photos of healthy skin as a control were selected. For each study category, two phases were adopted, characterized by an increasing number of files used for training. Finally, data were recorded and subjected to statistical analysis based on the following parameters: accuracy, precision, recall, F1-score, specificity.

**Results:**

Encouraging data have been observed, especially when compared with available scientific evidence. Numerous parameters were shown to be above the threshold attributed to the human limit, and in some fields, the highest predictive values to date were recorded.

**Discussion:**

Among the intrinsic limitations of the study, the limited data available emerged, especially for the algorithm training phase; moreover, it will be vital to proceed with the development of a specific software pre-trained on the basis of natural elements and secondarily directed to the recognition of the typical forensic characteristics. Among the strengths, however, it is possible to include the number of categories analyzed and the unprecedented use of an “intact skin” control category. In conclusion, the extension of the project to various research centers and the increase in the size of the sample adopted will allow the elimination of the emerged biases. This pilot study lays the groundwork for future multicenter research aimed at developing a robust and generalizable forensic AI model.

## 1 Introduction

Forensic ballistics can be briefly defined as the application of ballistic science for forensic purposes. The main task of this heterogeneous discipline is the reconstruction of events that caused a firearm GSW. It can be divided into different subcategories based on the specific area of analysis, including: internal ballistics (what happens inside the weapon at the moment of the shot’s explosion), external ballistics (which deals with the study of the projectile’s trajectory from the muzzle of the firearm to the target), and terminal ballistics (i.e., the analysis of the interaction between projectile and target). The latter, when referring to biological targets, is also defined as “wound ballistics” (1).

A full understanding of the mechanisms underlying Gunshot Wounds (GSW) on the human target implies a basic knowledge of the functioning of a weapon, i.e.: its structural characteristics (characteristics of the magazine, barrel), the type of ammunition used (e.g., single/multiple charge, composition, and shape), and the quality of propellant charge (referring to the type of primer and propellant powders used).

For the forensic pathologist, the interpretation of the injury patterns found at the crime scene represents a treacherous challenge. In fact, this field is characterized by multiple variables, including: the differential diagnosis between entry and exit skin holes from the projectile, the reconstruction of the “pathological” range of fire and, in general, the dynamics of the event and the attribution of the injury to a specific type of weapon versus another (2). These tasks are traditionally part of forensic tool mark analysis, where wound morphology is interpreted in relation to ballistic evidence. AI-based classification offers the potential to standardize this process, reduce subjective interpretation, and provide rapid preliminary assessments to guide further investigation.

Because of the complexity of the factors involved, a multidisciplinary approach is generally required, which involves collaboration between firearm examiners, radiologists, and laboratories in close cooperation.

Technologies based on Artificial Intelligence (AI), such as Machine Learning (ML) and computer vision, are extremely useful for solving clinical questions and/or criminal investigations, helping humans in the process of data analysis and interpretation.

However, we acknowledge that forensic wound analysis traditionally involves multi-modal data including ballistic characteristics, tissue deformation, and chemical residue. Our image-based approach is not intended to replace these methods but to offer a complementary tool for rapid, standardized visual classification. While traditional image processing techniques offer high interpretability for well-defined visual features, the variability and complexity of real-world GSWs often exceed the capabilities of rule-based methods. Deep Learning (DL) models, particularly CNNs, are better equipped to handle such non-linear patterns and morphological diversity.

Recently, there has been an increase in interest in making various open-source packages and commercial software available online. Up to date, AI in forensic science is applied to sectors such as sex and age identification based on DNA methylation assessment (3). Various tools have been applied to the analysis of haplogroups, simplifying and accelerating the classification of DNA samples, while others have been used to analyze short tandem repeat profiles, reducing misinterpretations (4). AI has been also employed on anthropometric data in the fields of forensic odontology and anthropology for the identification of human beings (5, 6); furthermore, it has demonstrated good suitability in the identification of taxonomic characters of diatoms (7).

Then, the application can be extended to the difficult attribution of causes of death. Other research has used this resource to determine the postmortem interval, and the results have sometimes exceeded human performance, significantly eliminating some residual biases (8).

Some researchers have explored the use of AI in the evaluation of GSWs, demonstrating that digitizing and analyzing fired bullets can help in the identification of firearms, as well as in the identification of entry and exit GSWs (9).

Our research group recently published a complementary study (10), assessing open-source software ChatGPT-4’s capabilities in forensic GSW classification using real-case images. While not based on DL, it reinforces the relevance of AI-assisted wound interpretation and highlights similar challenges in exit GSW recognition.

However, to achieve this goal, the most suitable technology among the neural networks existing today is the Convolutional Neural Network (CNN). Compared to the first neural model (the so-called “MP”) proposed by McCulloch and Pitts (11), the addition of multilayer perceptron architectures (12) with the development of backpropagation network (BP) systems, and finally, the introduction of the ImageNet Large Scale Visual Recognition Challenge (13), have led to the development of extremely advanced systems, capable of answering fine questions based on the identification, classification, and segmentation of recurring visual patterns (14).

Based on a sufficiently large training dataset, digital algorithms have demonstrated that they can classify images with accuracy comparable to a trained pathologist (15). Given the scarcity of large annotated forensic datasets (16), this study represents an initial step toward validating AI-based classification of GSWs using a limited but structured dataset.

Currently, a critical consultation of the main scientific databases has shown that only 4 articles have so far investigated the use of ML for the distinction of GSW characteristics. Between them, Oura equipe published two experiences in which different DL models were employed to estimate shooting distance from injury images. The first experience accounted for a dataset composed by 204 images from pig carcasses, achieving a 98% accuracy value (17), while the next one based his results on a total of 106 shotgun patterns from two discrete shooting distances recorded on blank white paper (accuracy performed: 94%) (18).

Three years later, in 2024 Cheng et al. (9) drew upon their local institution’s forensic archive, collecting 2,418 digital images from real cases; the objective of classifying GSWs between entrance or exit hole was reached with a registered accuracy of 87.99%.

Finally, Queiroz et al. (19) employed a dataset accounting for 2,551 images selected from a Brazilian forensic database. Aiming at distinguishing entrance wound morphological characteristics from the exit ones and estimating shooting distance, good accuracy values of 86.9% and 92.48% were, respectively, obtained. This result is actually the first and temporarily unique multi-purpose DL model-based study.

Up to date, scientific evidence comes from heterogeneous experiences when looking at cohorts and objectives, as well as softwares used and methodology for results analysis.

Despite the promising results achieved so far, the forensic context uniquely requires transparent and validated AI models, due to their role in judicial proceedings, where evidence can decisively influence verdicts. Therefore, the results achieved by cited studies (9, 17–19) represent a fundamental contribution in terms of proof of feasibility, but to be considered as preliminary experiences.

Therefore, the aim of this project is to explore the possibilities offered by the most modern technologies in relation to the recognition of the identifying characteristics of GSWs, taking further steps in scientific panorama.

The first step of this experiment was to collect multiple diagnostic questions in a single protocol, i.e., to expand the AI training field to all categories of GSWs that can be typed. The use of an open access package reflects the desire to provide data that can be replicated on a large scale and to prepare this research area for multicenter collaboration.

A secondary objective is to conceptualize a pilot software dedicated to the reporting of GSW images, with the future desired scope to be sufficiently accurate to be feasible in the judicial field.

## 2 Materials and methods

The study took place at the Institute of Legal Medicine of the University of Catania (hereinafter referred to as the “Institute”).

### 2.1 Data collection and organization

It was necessary to have photographic documents available. A subdivision of the image pools was implemented, using a distinction between training images and test images. Regarding the training images, the reasoning was conducted based on a question: do the images comprising the study pool have sufficient representativeness of the basic characteristics of the belonging labels? To this end, the adopted suitability criteria corresponded to the “ideal” characteristics of GSWs.

Therefore, in order to solve this problem and to provide examples that were as dogmatic as possible, a selection of images was made from a specialized illustrated atlas on forensic ballistics (6).

In compliance with the color range and file size requirements subsequently imposed on the test images (artificial lighting, RGB color profile, minimum resolution), it was possible to preliminarily extract 100 high-resolution images, all characterized by the presence of a metric reference (graduated object). This is a characteristic often present in photographs relating to forensic investigations and part of the Institute’s operating framework.

Subsequently, and in order to build a pool of test images, a search was conducted within the computer archives owned by the Institute. The selection criteria for the found images were as follows:

- Subject: cases of GSWs;
- Origin: Nikon D750 camera with proprietary lens, or Nikon D850 with proprietary lens;
- Lighting: artificial light, integrated flash of the device;
- Color profile: sRGB IEC61966-2.1;
- Alpha channel: no;
- Minimum dimensions (of the detail): 3000 × 2000 pixels.

The application of these outlined criteria allowed for the selection of 40 photographic files with homogeneous characteristics. In this phase, it was essential to have a proprietary archive of photographs relating to the forensic activity conducted over the years.

In any case, in compliance with the minimum resolution selected, any direct reference to the identity of the immortalized subjects and the surrounding environment was carefully eliminated when other than the autopsy room of the Institute of Forensic Medicine of Catania.

Subsequently, the same technical criteria guided the random extraction of 40 photographic files featuring human skin free from pathological alterations, included in the study as a control.

As expected on the basis of scientific evidence (20), the subdivision of the unpublished images into study categories (labels) generated an imbalance in terms of category representation (certain types of shooting dynamics are generally rarer than others).

To overcome this problem, further data augmentation was performed, i.e., expanding the number of database files based on techniques aimed at improving the performance of learning models by increasing the virtual size and diversity of training data without needing a larger data pool (21, 22).

**Figure 1:**
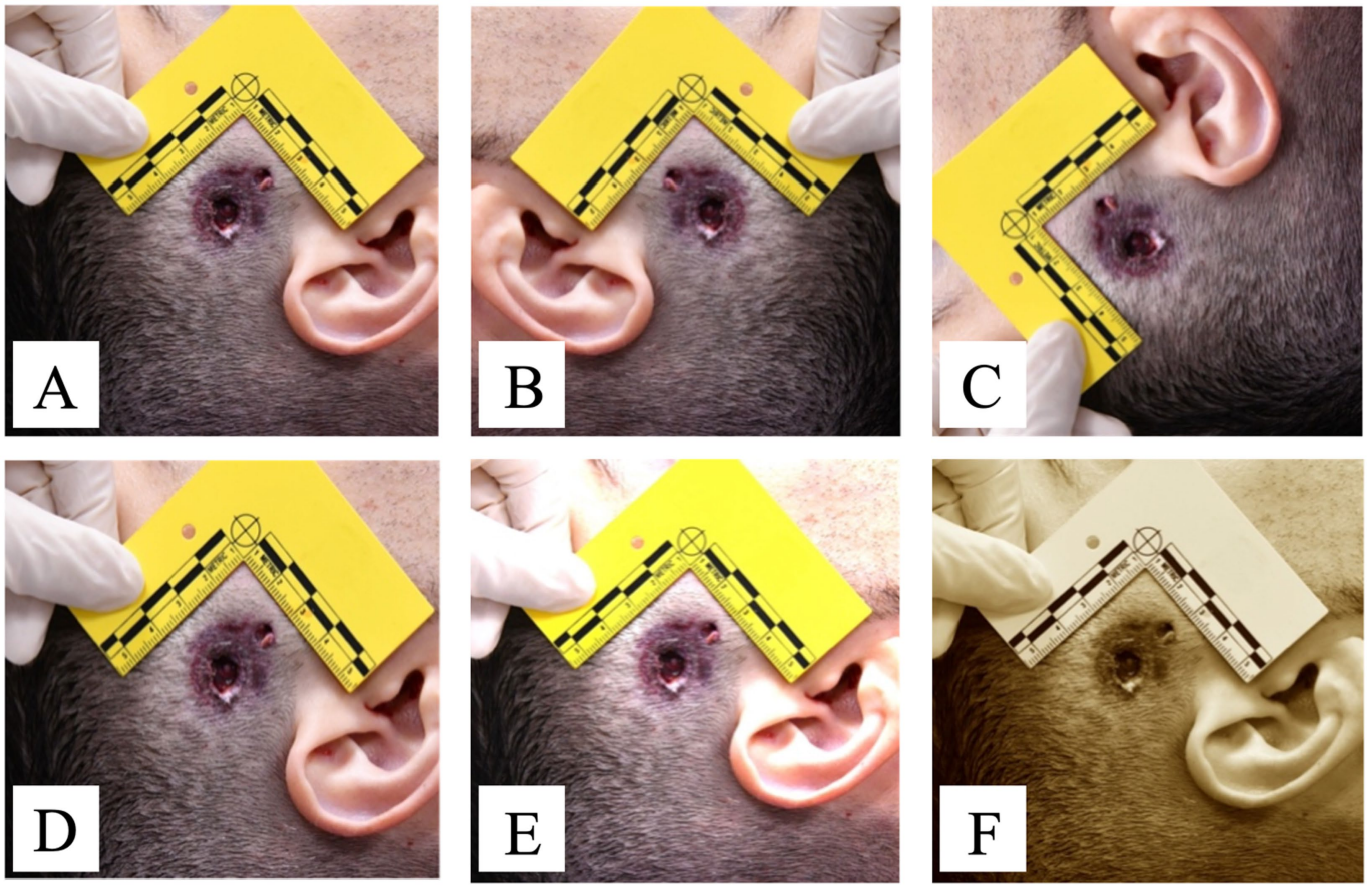
Example of a contact shot GSW photo **(A)** and related copies subjected to modifications for data augmentation: **(B)** symmetrical flip on the major axis; **(C)** 90-degree rotation; **(D)** random cropping around the area of interest; **(E)** color blurring; **(F)** application of the sepia filter. GSW, gunshot wound.

For this reason, the pool of 40 photos relating to the Institute’s case history was manually manipulated for data augmentation, by executing the following systematic modifications (Figure 1):

- Copy 1: symmetrical flip on the major axis;
- Copy 2: 90-degree rotation;
- Copy 3: random cropping around the area of interest;
- Copy 4: color blurring;
- Copy 5: application of the sepia filter.

In this way, it was possible to reach a total number of 240 test photos as the definitive data pool admitted to the study.

The choice of these techniques has been shared with other authors in the scientific landscape (9, 17). Moreover, we aimed to avoid excessive distortion of anatomical and pathological elements that could influence the algorithm. In this way, keeping shapes and contrast the most faithful to the original pictures represented the key to avoid recognition biases, as well as keeping images copy number as low as possible. On the other hand, casual randomization of image administration order ensured a good sample variability (overfitting risk assessment).

Therefore, the 5 basic variations chosen for the data augmentation of this study are the simplest, most controllable, and systematic. Still in terms of avoiding overfitting, the small amount of data available has not prevented us from having a fair amount of morphological variability in the proposed subjects: the original files in fact presented notable heterogeneity in terms of skin pigmentation, topographical distribution of the lesions, presence or absence of blood material, epidermal appendages (e.g., moles, hair, nipples).

Finally, to prevent bias and ensure model generalization, all augmented images were used exclusively for training purposes. The test set consisted solely of original, non-augmented images from the forensic archive and control skin samples. No image or its augmented copy was present in both training and testing sets.

### 2.2 Employed tools

Based on preliminary data collected previously using a commonly used “generative AI,” the same experimental model was applied using an open-source AI software for recognition (Lobe.ai) (23).

The selected tool is characterized by a high degree of specialization in learning aimed at identifying graphic elements, as well as a highly intuitive interface. “Lobe.ai” is an AutoML program, corresponding to a generalized research concept, with specialized search algorithms to find the optimal solutions for each component of the ML pipeline. In other words, this means creating AI content without manual coding.

In the case of Lobe, the use of the algorithm is specifically directed at image classification: so, briefly, it is sufficient to provide Lobe with a set of images with labels to automatically find by itself the optimal classification model. Furthermore, this program is completely free: this is an undoubted advantage because there will not be financial outlays for license purchases in case of multicentric expansion of the research.

Lobe.ai allows the use of two different algorithms, one optimized for speed [MobileNetV2 (24)] and one dedicated to accuracy [ResNet-50 V2 (25)]. A parallel test of the two algorithms showed a negligible increase in response time by the second compared to the first, so we employed exclusively ResNet-50 V2 in the current research.

Ultimately, ResNet-50 V2 was chosen due to its superior performance in prior forensic studies and its availability within the Lobe AI platform. However, we acknowledge that a broader benchmarking against other architectures and traditional image processing methods would provide in the future a more comprehensive evaluation of different models’ effectiveness.

### 2.3 Experimental model

The study was divided into work categories, and the following fields and subcategories (“labels”) were identified:

- Field 1: GSWs – healthy skin.
- Field 2: entry GSWs – exit GSWs – healthy skin.
- Field 3: close-range shot – intermediate-range shot – long-range shot – healthy skin.
- Field 4: single charge ammunition – multiple charge ammunition – healthy skin.

Conceptually, four consecutive phases were identified for each experimental session:

- Labeling and training (machine learning);
- Validation;
- Testing;
- Data recording.

Specifically, each field was subject to two distinct study sessions, characterized by an increasing number of images provided for training. Specifically, the following order of files was used:

- Field 1 - Phase 1: 5 GSW files – 5 healthy skin files. Total: 10 files.
- Field 1 - Phase 2: 15 GSW files – 15 healthy skin files. Total: 30 files.
- Field 2 - Phase 1: 5 entry GSW files – 5 exit GSW files - 5 healthy skin files. Total: 15 files.
- Field 2 - Phase 2: 15 entry GSW files – 15 exit GSW files - 15 healthy skin files. Total: 45 files.
- Field 3 - Phase 1: 5 close-range GSW files – 5 intermediate-range GSW files – 5 long-range GSW files – 5 healthy skin files. Total: 20 files.
- Field 3 - Phase 2: 11 close-range GSW files – 11 intermediate-range GSW files – 11 long-range GSW files – 11 healthy skin files. Total: 44 files.
- Field 4 - Phase 1: 5 single charge ammunition GSW files – 5 multiple charge ammunition GSW files - 5 healthy skin files. Total: 15 files.
- Field 4 - Phase 2: 15 single charge ammunition GSW files – 15 multiple charge ammunition GSW files - 15 healthy skin files. Total: 45 files.

The decision to subject the algorithm to two distinct study phases for each designated field was dictated by the desire to verify the incidence of more extensive training on the final result.

Furthermore, during the testing phase, the order of file administration was as follows: first, the image pools relating to the individual categories were subjected to random mixing, in order to avoid pitfalls as administering consecutive files deriving from the same native one (as a result of data augmentation),; subsequently, the image delivery followed an alternating order depending on the specific Phase’s number of categories.

The decision not to submit to the algorithm consecutive files of the same category was taken due to the evolutionary nature of the AI’s recognition capacity and the concrete risk of overfitting data. Unfortunately, limitedness of our sample prevented us from the application of more accurate cross-validation techniques [e.g., stratification, K-folds (26)].

This represents an actual dropout of the present research, that we aim to eliminate in the future by expanding training and testing datasets.

To allow continuous monitoring of the algorithm’s performance during the testing phase, the fourth phase (data recording) occurred simultaneously with the third (testing).

The emerging data were recorded on Microsoft Excel tables specifically prepared for table recording of the predictions advanced by the algorithm, according to a tripartite subdivision:

- Correct answer (green dot);
- Uncertain answer, corresponding to the attribution of a label, but according to a degree of certainty of the algorithm lower than 100% (yellow dot);
- Wrong answer (red dot).

The schematization of the data obtained was essential not only for the subdivision of the produced answers: in fact, the recording of the response rates was carried out by dividing the register into thirds (first third, second third, last third) and finally proceeding to the counting of the results obtained overall.

### 2.4 Statistical analysis

Statistical analysis was conducted to evaluate the effectiveness of the present machine learning model. The following parameters were calculated for each category analyzed: Accuracy, Precision, Recall, F1-Score, and Specificity; relative formulas and meanings are reported in Table 1 (27). These indicators allow a comprehensive evaluation of the model’s performance in terms of predictive capacity and reliability.

**Table 1:** List of parameters employed for statistical analysis, enriched with relative formulas and explanation.

Definition	Formula	Implications
Accuracy	$\frac{\mathrm{TP}+\mathrm{TN}}{\mathrm{TP}+\mathrm{TN}+\mathrm{FP}+\mathrm{FN}}$	Reflection of the model’s ability to correctly classify data points. As a con, it treats all classes as equally important and looks at all correct predictions.
Precision	$\frac{\mathrm{TP}}{\mathrm{TP}+\mathrm{FP}}$	It measures how often a machine learning model correctly predicts the positive class. It is useful when the cost of a false positive is high.
Recall	$\frac{\mathrm{TP}}{\mathrm{TP}+\mathrm{FN}}$	It measures how often a machine learning model correctly identifies positive instances (true positives) from all the actual positive samples in the dataset. The term “sensitivity” is more commonly used in medical and biological research.
F1-score	$\frac{\text{Precision}\times \text{Recall}}{\text{Precision}+\text{Recall}}$	It is the harmonic mean of Precision and Recall, offering a single measure that accounts for both metrics. It is especially useful when dealing with imbalanced datasets.
Specificity	$\frac{\mathrm{TN}}{\mathrm{TN}+\mathrm{FP}}$	It measures the model’s ability to identify true negatives. In other words, it depicts the probability of a negative test given that the patient does not have the disease.

TP, true positives; FP, false positives; TN, true negatives; FN, false negatives.

The data relating to correct answers (green dot), uncertain answers (yellow dot), and incorrect answers (red dot) were aggregated for each field and experimental session. The parameters defined above were calculated using the classification frequencies for GSWs and healthy skin (Field 1), entry and exit GSWs (Field 2), shooting distances (Field 3), and ammunition types (Field 4).

The analyses were performed using Microsoft Excel software and cross-validations on dedicated statistical platforms.

## 3 Results

This study was conducted during the period of September–December 2024 among the Institute of Legal Medicine of the University of Catania.

Following the recording of the data obtained from the algorithm’s testing phase, prior to statistical analysis, the trend of the correct answer rate was observed by dividing the results into homogeneous subgroups. By this way, it was possible to observe the progress in terms of accuracy during the testing phases.

### 3.1 Field 1

**Figure 2:**
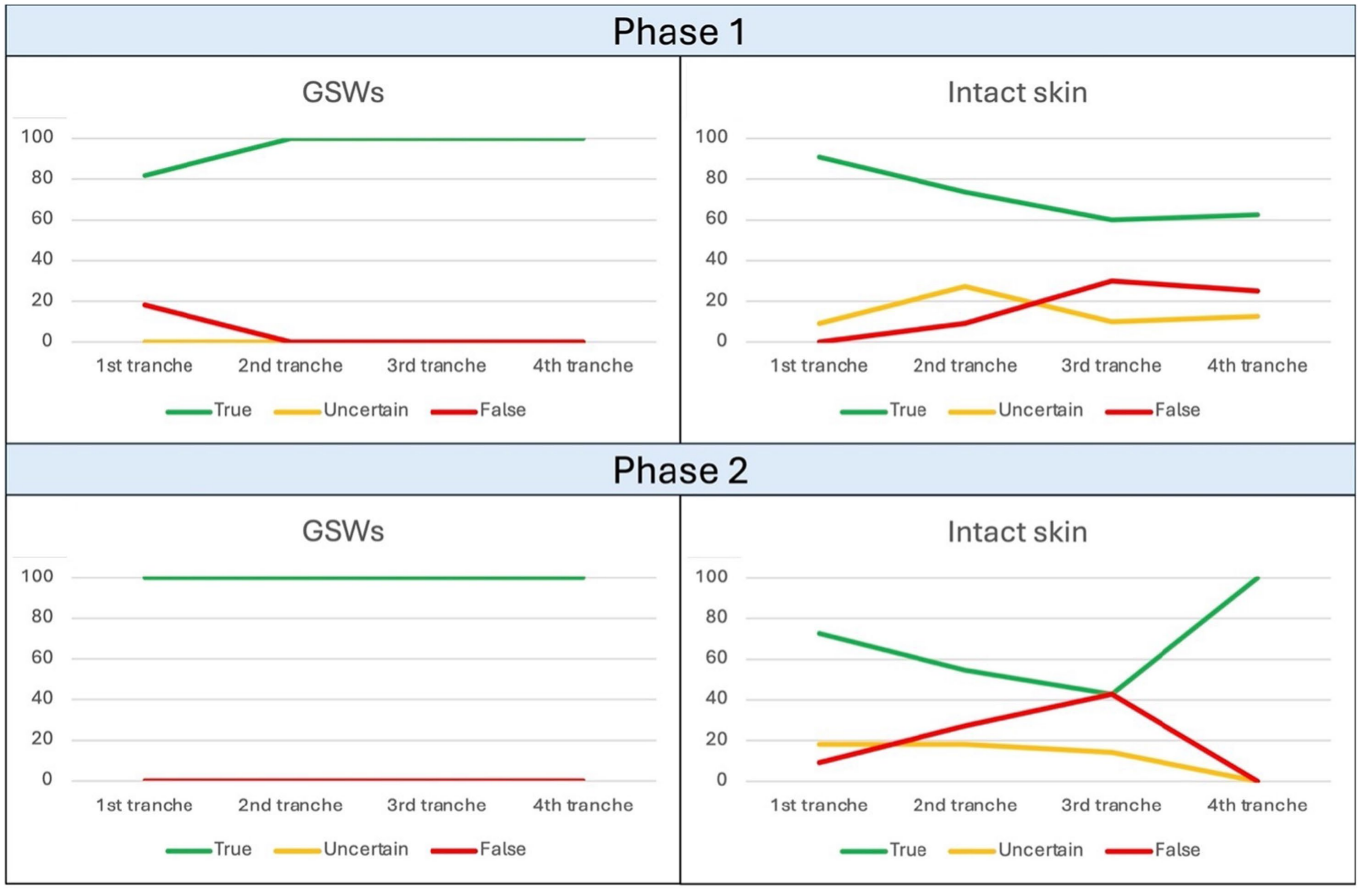
Trend of observed responses in Field 1. The ordinate axis reports percent values from 0 to 100, while abscissa axis depicts sequential tranches of data administration to the algorithm.

For the first phase of the study related to Field 1, 10 files were used for training, and a trend of the response rate was observed as illustrated in Figure 2, corresponding to a progressive increase in the rate of correct answers for the “GSW” category (final value 100%) and, conversely, a progressive decrease in the rate of correct answers for the “intact skin” category. Within Table 2, it is instead possible to observe the results in percentage terms.

**Table 2:** Analysis of the rate of correct, uncertain, and incorrect answers at the end of the test (Phase 1, Field 1).

Subject	Evaluation	% 1st tranche	% 2nd tranche	% 3rd tranche	% 4th tranche	*Δ* 1–2 tranche	Results	Δ 2–3 tranche	Results	Δ 3–4 tranche	Results	Δ 1–4 tranche	Results
GSW	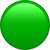	81.8	100	100	100	18.2	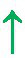	0	=	0	=	18.2	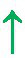
	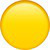	0	0	0	0	=	0	=	0	=	0	0	=
	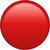	18.2	0	0	0	18.2	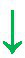	0	=	0	=	18.2	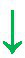
Intact skin	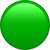	90.9	73.6	60	62.5	17.3	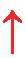	13.6	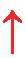	2.5	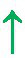	28.4	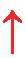
	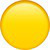	9.1	27.3	10	12.5	18.2	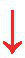	17.3	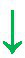	2.5	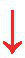	3.4	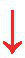
	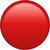	0	9.1	30	25	9.1	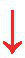	20.9	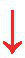	5	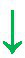	25	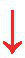

GSW, gunshot wound.

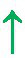
: positive response in terms of increase in correct answers rate.

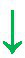
: positive response in terms of decrease in correct answers rate.

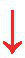
: negative response in terms of increase in correct answers rate.

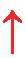
: negative response in terms of decrease in correct answers rate.

For the second phase of the study related to Field 1, 30 files were used for training, and a trend of the response rate was observed corresponding to a plateau of 100% correct answers for the “GSW” category, while the “intact skin” category observed a cusp pattern with an initial decrease in correct answers followed by a new increase (Figure 2).

Within Table 3, it is instead possible to observe the results in percentage terms; due to the overall 28.4% decrease of correct answers from intact skin category within sequential phases, it could be assumed an overfitting issue which can be avoided in future with larger datasets.

**Table 3:** Analysis of the rate of correct, uncertain, and incorrect answers at the end of the test (Phase 2, Field 1).

Subject	Evaluation	% 1st tranche	% 2nd tranche	% 3rd tranche	% 4th tranche	Δ 1–2 tranche	Results	Δ 2–3 tranche	Results	Δ 3–4 tranche	Results	Δ 1–4 tranche	Results
GSW	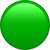	100	100	100	100	0	=	0	=	0	=	0	=
	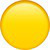	0	0	0	0	0	=	0	=	0	=	0	=
	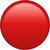	0	0	0	0	0	=	0	=	0	=	0	=
Intact skin	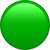	90.9	73.6	60	62.5	17.3	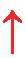	13.6	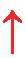	2.5	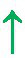	28.4	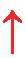
	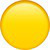	9.1	27.3	10	12.5	18.2	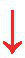	17.3	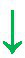	2.5	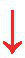	3.4	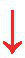
	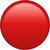	0	9.1	30	25	9.1	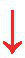	20.9	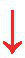	5	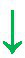	25	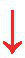

GSW, gunshot wound.

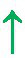
: positive response in terms of increase in correct answers rate.

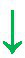
: positive response in terms of decrease in correct answers rate.

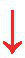
: negative response in terms of increase in correct answers rate.

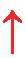
: negative response in terms of decrease in correct answers rate.

### 3.2 Field 2

**Figure 3:**
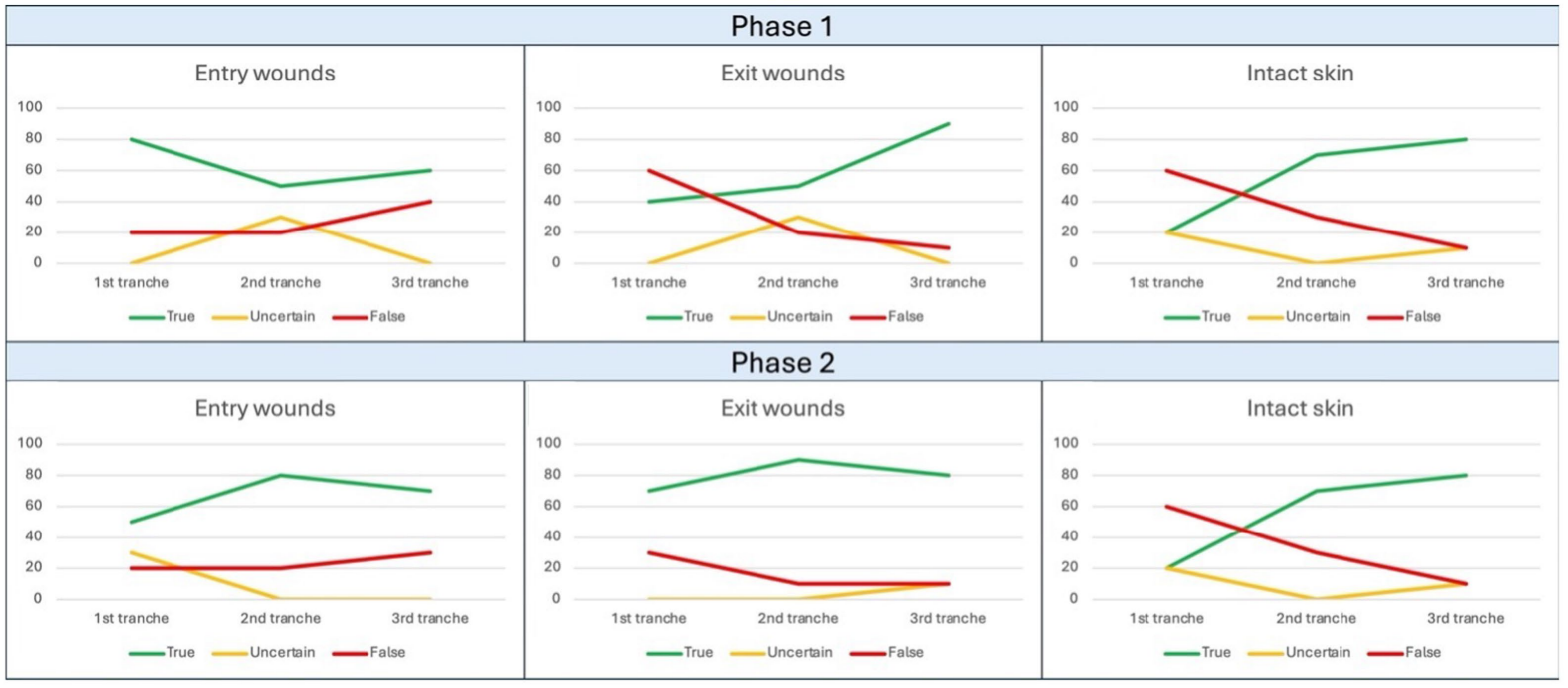
Trend of observed responses in Field 2. The ordinate axis reports percent values from 0 to 100, while abscissa axis depicts sequential tranches of data administration to the algorithm.

For the first phase of the study related to Field 2, 15 files were used for training, and a progressive decrease in correct answers was observed exclusively for the “entry GSW” category, while the others were characterized by a fluctuating pattern (Figure 3). Within Table 4, it is instead possible to observe the results in percentage terms, which quantify the same phenomenon. In particular, it can be observed a terminal opposite trend between correct and false answers, without variations among the uncertain conclusions.

**Table 4:** Analysis of the rate of correct, uncertain, and incorrect answers at the end of the test (Phase 1, Field 2).

Subject	Evaluation	% 1st tranche	% 2nd tranche	% 3rd tranche	Δ 1–2 tranche	Results	Δ 2–3 tranche	Results	Δ 1–3 tranche	Results
Entry GSW	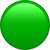	80	50	60	30	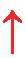	10	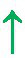	20	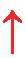
	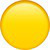	0	30	0	30	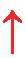	30	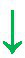	0	=
	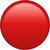	20	20	40	0	=	20	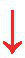	20	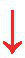
Exit GSW	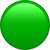	40	70	90	30	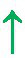	20	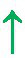	50	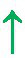
	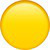	0	0	0	0	=	0	=	0	=
	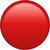	60	30	10	30	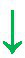	20	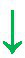	50	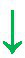
Intact skin	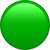	20	70	80	50	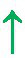	10	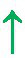	60	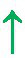
	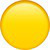	20	0	10	20	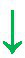	10	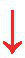	10	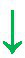
	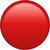	60	30	10	30	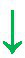	20	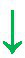	50	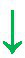

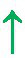
: positive response in terms of increase in correct answers rate.

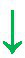
: positive response in terms of decrease in correct answers rate.

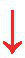
: negative response in terms of increase in correct answers rate.

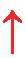
: negative response in terms of decrease in correct answers rate.

For the second phase of the study related to Field 2, 45 files were used for training, and it was observed how this time the two “pathological” categories, corresponding to GSWs, followed very similarly a pattern of initial improvement followed by a final worsening (Figure 3) quantifiable as 20% for entry GSWs and as 10% for both exit GSWs and intact skin images. Then, within Table 5, it is instead possible to observe the results in percentage terms.

**Table 5:** Analysis of the rate of correct, uncertain, and incorrect answers at the end of the test (Phase 2, Field 2).

Subject	Evaluation	% 1st tranche	% 2nd tranche	% 3rd tranche	Δ 1–2 tranche	Results	Δ 2–3 tranche	Results	Δ 1–3 tranche	Results
Entry GSW	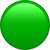	50	80	70	30	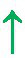	10	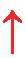	20	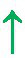
	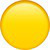	30	0	0	30	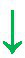	0	=	30	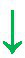
	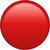	20	20	30	0	=	10	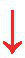	10	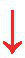
Exit GSW	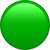	70	90	80	20	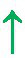	10	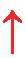	10	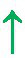
	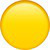	0	0	10	0	=	10	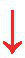	10	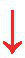
	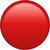	30	10	10	20	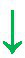	0	=	20	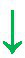
Intact skin	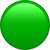	70	80	60	10	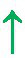	20	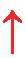	10	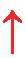
	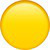	0	10	0	10	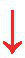	10	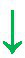	0	=
	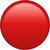	30	10	40	20	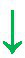	30	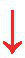	10	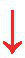

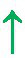
: positive response in terms of increase in correct answers rate.

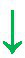
: positive response in terms of decrease in correct answers rate.

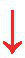
: negative response in terms of increase in correct answers rate.

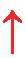
: negative response in terms of decrease in correct answers rate.

### 3.3 Field 3

**Figure 4:**
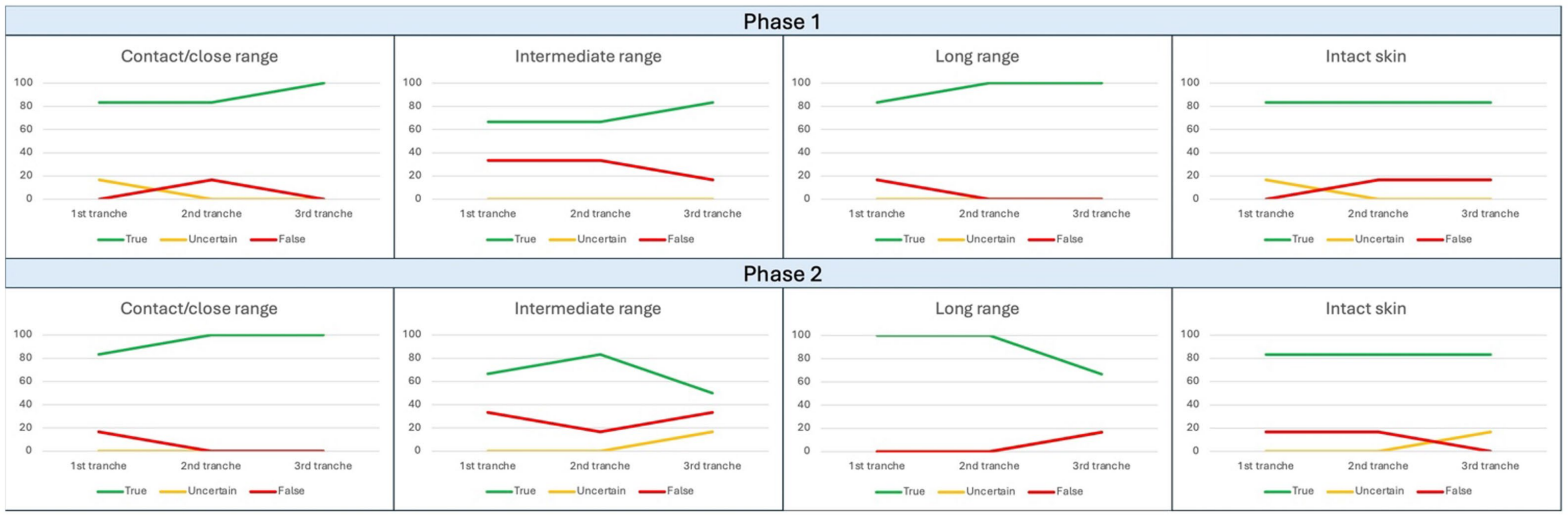
Trend of observed responses in Field 3. The ordinate axis reports percent values from 0 to 100, while abscissa axis depicts sequential tranches of data administration to the algorithm.

For the first phase of the study related to Field 3, 20 files were used for training, and the results proved to be extremely encouraging: at the end of the experiment, the distance categories “contact/close range” and “long range” showed a correct answer rate of 100%, while the “intermediate range” category progressively increased its value to exceed 80% as well as for the intact skin category which however showed no variation during the experiment (Figure 4). Within Table 6, it is instead possible to observe the results in percentage terms.

**Table 6:** Analysis of the rate of correct, uncertain, and incorrect answers at the end of the test (Phase 1, Field 3).

Subject	Evaluation	% 1st tranche	% 2nd tranche	% 3rd tranche	Δ 1–2 tranche	Results	Δ 2–3 tranche	Results	Δ 1–3 tranche	Results
Contact-close range shot	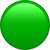	83.3	83.3	100	0	=	16.7	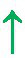	16.7	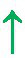
	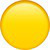	16.7	0	0	16.7	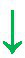	0	=	16.7	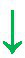
	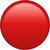	0	16.7	0	16.7	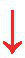	16.7	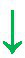	0	=
Intermediate range shot	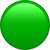	66.6	66.6	83.3	0	=	16.7	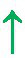	16.7	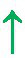
	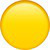	0	0	0	0	=	0	=	0	=
	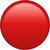	33.4	33.4	16.7	0	=	16.7	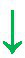	16.7	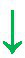
Long range shot	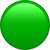	83.3	100	100	16.7	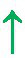	0	=	16.7	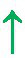
	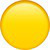	0	0	0	0	=	0	=	0	=
	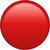	16.7	0	0	16.7	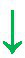	0	=	16.7	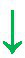
Intact skin	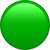	83.3	83.3	83.3	0	=	0	=	0	=
	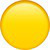	16.7	0	0	16.7	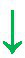	0	=	16.7	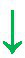
	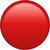	0	16.7	16.7	16.7	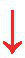	0	=	16.7	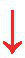

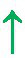
: positive response in terms of increase in correct answers rate.

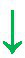
: positive response in terms of decrease in correct answers rate.

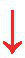
: negative response in terms of increase in correct answers rate.

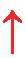
: negative response in terms of decrease in correct answers rate.

For the second phase of the study related to Field 3, 44 files were used for training; unlike Phase 1, the only categories which showed an increasing trend in the correct answer rate was the “contact-close range of fire” group, as will be analyzed in the Discussion section (Figure 4). Within Table 7, it is instead possible to observe the results in percentage terms and to compare trends across subsequential tranches.

**Table 7:** Analysis of the rate of correct, uncertain, and incorrect answers at the end of the test (Phase 2, Field 3).

Subject	Evaluation	% 1st tranche	% 2nd tranche	% 3rd tranche	Δ 1–2 tranche	Results	Δ 2–3 tranche	Results	Δ 1–3 tranche	Results
Contact-close range shot	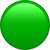	83.3	100	100	16,7	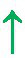	0	=	16.7	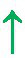
	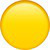	0	0	0	0	=	0	=	0	=
	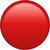	16.7	0	0	16.7	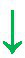	0	=	16.7	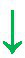
Intermediate range shot	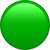	66.6	83.3	50	16.6	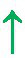	53.3	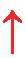	16.6	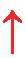
	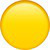	0	0	16.7	0	=	16.7	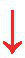	16.7	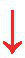
	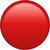	33.4	16.7	33.3	16.7	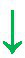	16.7	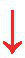	0	=
Long range shot	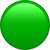	100	100	66.6	0	=	33.3	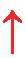	33.3	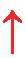
	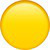	0	0	16.7	0	=	16.7	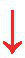	16.7	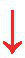
	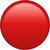	0	0	16.7	0	=	16.7	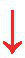	16.7	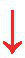
Intact skin	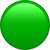	83.3	83.3	83.3	0	=	0	=	0	=
	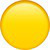	0	0	16.7	0	=	16.7	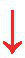	16.7	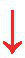
	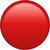	16.7	16.7	0	0	=	16.7	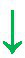	16.7	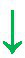

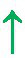
: positive response in terms of increase in correct answers rate.

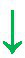
: positive response in terms of decrease in correct answers rate.

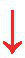
: negative response in terms of increase in correct answers rate.

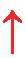
: negative response in terms of decrease in correct answers rate.

### 3.4 Field 4

**Figure 5:**
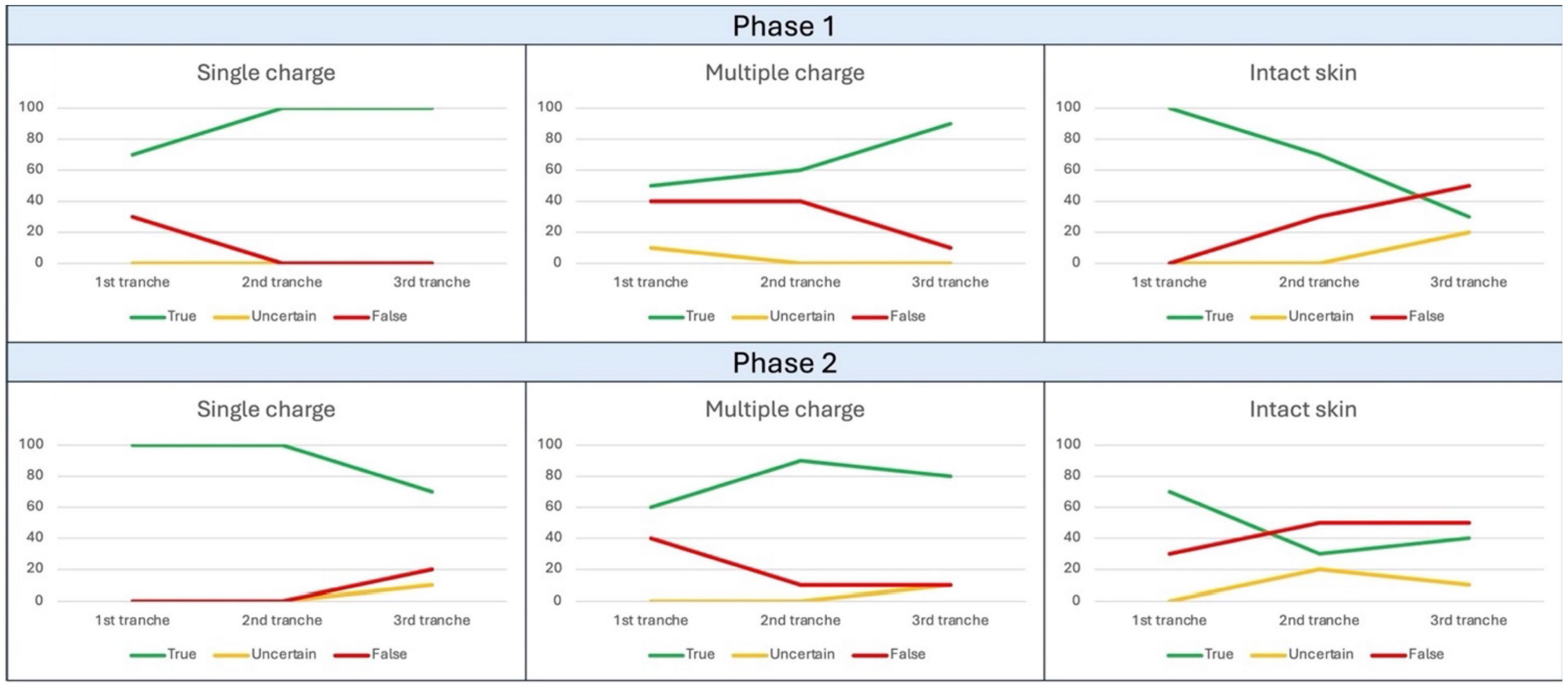
Trend of observed responses in Field 4. The ordinate axis reports percent values from 0 to 100, while abscissa axis depicts sequential tranches of data administration to the algorithm.

For the first phase of the study related to Field 4, 15 files were used for training. The decreasing pattern observed for the “intact skin” category appears apparently inexplicable, which went from an initial correct answer rate of 100% to a final 30% (Figure 5). Within Table 8, it is instead possible to observe the results of every category in percentage terms.

**Table 8:** Analysis of the rate of correct, uncertain, and incorrect answers at the end of the test (Phase 2, Field 4).

Subject	Evaluation	% 1st tranche	% 2nd tranche	% 3rd tranche	Δ 1–2 tranche	Results	Δ 2–3 tranche	Results	Δ 1–3 tranche	Results
Single charge ammunition	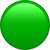	70	100	100	30	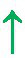	0	=	30	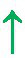
	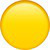	0	0	0	0	=	0	=	0	=
	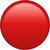	30	0	0	30	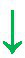	0	=	30	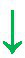
Multiple charge ammunition	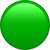	50	60	90	10	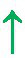	30	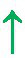	40	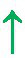
	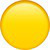	10	0	0	10	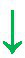	0	=	10	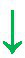
	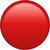	40	40	10	0	=	30	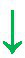	30	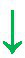
Intact skin	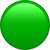	100	70	30	30	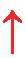	40	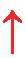	70	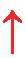
	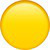	0	0	20	0	=	20	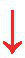	20	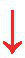
	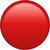	0	30	50	30	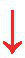	20	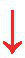	50	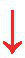

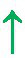
: positive response in terms of increase in correct answers rate.

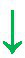
: positive response in terms of decrease in correct answers rate.

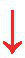
: negative response in terms of increase in correct answers rate.

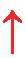
: negative response in terms of decrease in correct answers rate.

For the second phase of the study related to Field 4, 45 files were used for training; once again, at the end of the experiment, it was possible to denote a decremental pattern of correct answers not only for intact skin category, but also for the “single charge ammunition GSWs” one (Figure 5). Within Table 9, it is instead possible to observe the results in percentage terms.

**Table 9:** Analysis of the rate of correct, uncertain, and incorrect answers at the end of the test (Phase 2, Field 4).

Subject	Evaluation	% 1st tranche	% 2nd tranche	% 3rd tranche	Δ 1–2 tranche	Results	Δ 2–3 tranche	Results	Δ 1–3 tranche	Results
Single charge ammunition	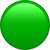	100	100	70	0	=	30	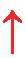	30	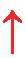
	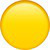	0	0	10	0	=	10	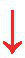	10	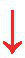
	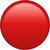	0	0	20	0	=	20	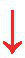	20	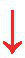
Multiple charge ammunition	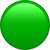	60	90	80	30	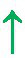	10	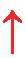	20	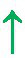
	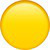	0	0	10	0	=	10	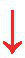	10	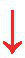
	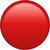	40	10	10	30	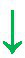	0	=	30	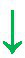
Intact skin	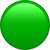	70	30	40	40	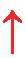	10	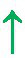	30	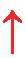
	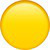	0	20	10	20	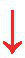	10	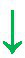	10	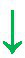
	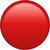	30	50	60	20	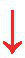	10	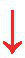	30	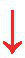

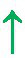
: positive response in terms of increase in correct answers rate.

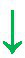
: positive response in terms of decrease in correct answers rate.

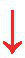
: negative response in terms of increase in correct answers rate.

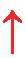
: negative response in terms of decrease in correct answers rate.

### 3.5 Statistical analysis

The results of the tested model were evaluated according to standard parameters. Due to the poor representation of the “uncertain answers” category, it was not possible to proceed with a multivariate analysis of the data. For this reason, in the interpretation of the data in this paper (to be considered preliminary to a future expansion of the database used), uncertain answers were assimilated to false ones, reducing the data count to binomial categories.

In the future, datasets expansion will allow a more accurate analysis of the results, implementing the degree of uncertainty of the model in terms of quality and quantity.

**Figure 6:**
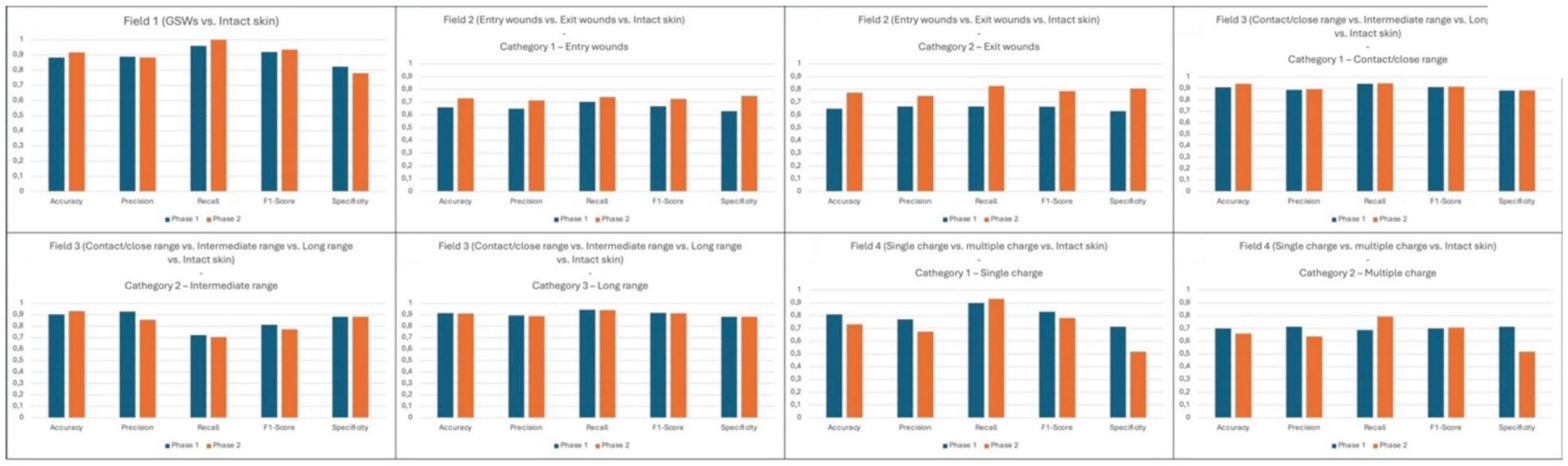
Visual representation of statistical analysis from all fields. The ordinate axis reports the 0–1 range chosen to standardize results obtained from our performance metrics, which are reported on abscissa axis.

The numerical values of the interpolation conducted are represented within Tables 10–17 and visually represented within Figure 6.

**Table 10:** Results of the statistical analysis related to Field 1 (GSW vs. intact skin).

Statisticalparameter	Phase 1 (10 files used for training)	Phase 2 (30 files used for training)
Accuracy	0.883	0.917
Precision	0.888	0.883
Recall	0.96	1
F1-score	0.92	0.936
Specificity	0.823	0.781

GSW, gunshot wound.

**Table 11:** Results of the statistical analysis related to Field 2 (entry GSWs vs. intact skin).

Statisticalparameter	Phase 1 (15 files used for training)	Phase 2 (45 files used for training)
Accuracy	0.66	0.73
Precision	0.65	0.714
Recall	0.703	0.74
F1-score	0.668	0.726
Specificity	0.629	0.75

**Table 12:** Results of the statistical analysis related to Field 2 (exit GSWs vs. intact skin).

Statisticalparameter	Phase 1 (15 files used for training)	Phase 2 (45 files used for training)
Accuracy	0.649	0.775
Precision	0.666	0.75
Recall	0.666	0.827
F1-score	0.665	0.786
Specificity	0.629	0.807

**Table 13:** Results of the statistical analysis relating to Field 3 (Contact/close range shot vs. intact skin).

Statisticalparameter	Phase 1 (20 file impiegati per l’addestramento)	Phase 2 (44 file impiegati per l’addestramento)
Accuracy	0.911	0.941
Precision	0.888	0.894
Recall	0.941	0.944
F1-score	0.913	0.917
Specificity	0.882	0.882

**Table 14:** Results of the statistical analysis related to Field 3 (intermediate range shot vs. intact skin).

Statisticalparameter	Phase 1 (20 files used for training)	Phase 2 (44 files used for training)
Accuracy	0.903	0.831
Precision	0.928	0.857
Recall	0.722	0.705
F1-score	0.812	0.773
Specificity	0.882	0.882

**Table 15:** Results of the statistical analysis related to Field 3 (long range shot vs. intact skin).

Statisticalparameter	Phase 1 (20 files used for training)	Phase 2 (44 files used for training)
Accuracy	0.914	0.911
Precision	0.894	0.888
Recall	0.944	0.941
F1-score	0.917	0.913
Specificity	0.882	0.882

**Table 16:** Results of the statistical analysis related to Field 4 (single charge ammunition vs. intact skin).

Statisticalparameter	Phase 1 (15 files used for training)	Phase 2 (45 files used for training)
Accuracy	0.81	0.732
Precision	0.771	0.675
Recall	0.9	0.931
F1-score	0.83	0.782
Specificity	0.714	0.518

**Table 17:** Results of the statistical analysis related to Field 4 (multiple charge ammunition vs. intact skin).

Statisticalparameter	Phase 1 (15 files used for training)	Phase 2 (45 files used for training)
Accuracy	0.701	0.66
Precision	0.714	0.638
Recall	0.689	0.793
F1-score	0.701	0.707
Specificity	0.714	0.518

## 4 Discussion

The results obtained from the chosen algorithm have proven to be promising, both in terms of responsiveness to training and correct answer rate.

As an introduction, in fact, the non-inferiority objective of the Lobe software was set as the predictive value emerging from the scientific experiences so far in the literature (9, 17–19), considering the experience of Queiroz Nogueira Lira et al. (19) as the most significant so far. Queiroz team obtained a predictive value of 86.9% for the “GSW type” (entry/exit) and 92.5% for the “shooting distance,” accounting on a very large dataset (2,551 images) but considering less classification variables than the present work.

As a yardstick, values above 85% have been previously considered close to human performance, referred as the ability to discern the salient characteristics of a GSW by the forensic pathologist with proven field experience (9).

It is necessary to specify that, in the cited experience, the authors used numerous DL softwares (12 in total) but that, systematically, the most reliable results were derived from ResNet. It is therefore an element of absolute value for the present investigation that all the detections were carried out using the ResNet software in its version updated in 2024, namely the V2 (25).

Nonetheless, the absence of comparative testing with alternative models represents a limitation. Future studies should include benchmarking against other CNN architectures (e.g., EfficientNet, DenseNet) and traditional image analysis techniques to assess relative performance and interpretability.

Also Cheng et al. (9) have recorded encouraging values (accuracy equal to 87.99%), even on the basis of a large case history (2,418 images) but lacking a higher categorical complexity: in fact, only entry and exit GSWs were investigated at the time.

The Oura team (17, 18), instead, obtained significantly higher values but in absence of a true human target.

It is clear from these premises the exploratory nature of this study introduces novel elements to the forensic AI domain. Beyond classification metrics, the practical forensic utility of this approach lies in its ability to assist pathologists in real-time decision-making. For example, in cases with ambiguous wound morphology or degraded tissue, AI can offer a reproducible and quantifiable second opinion. This is particularly valuable in judicial contexts where expert testimony must be supported by objective data. Nonetheless, we recognize the importance of interpretability in forensic applications.

Future work will include comparative studies with traditional image analysis techniques and the integration of explainable AI (XAI) frameworks to enhance transparency and trust in model outputs. It is, in fact, characterized by the following peculiarities:

- Extreme scarcity of pre-existences in the scientific literature;
- First study related to DL to use the ResNet V2 software [the predecessor, ResNet, had already proven better than the competitors in the comparative study of Queiroz Nogueira Lira et al. (19)];
- First study in Italy to associate cognitive neural networks to forensic ballistics;
- First study to consider as a category the “single/multiple charge ammunition GSW” distinction;

To date, ours constitutes the study with more ballistic parameters simultaneously implemented. Therefore, evaluating the predictability of the individual study categories at the end of the investigation (Table 18), it is possible to advance some considerations.

**Table 18:** Analysis of correct answers percentage value relative to all phases of the present study.

	Phase of study	GSWs^*,Δ^	Healthy skin
Field 1	Phase 1	96	70
	Phase 2	100	67.5

		Entry GSWs	Exit GSWs	Healthy skin
Field 2	Phase 1	63.3	66.6	56.6
	Phase 2	66.6	80	60

		Close range shot	Intermediate range shot	Long range shot	Healthy skin^†^
Field 3	Phase 1	88.9	72.2	94.4	83.3
	Phase 2	94.4	66.7	88.9	83.3

		Single charge ammunition	Multiple charge ammunition	Healthy skin
Field 4	Phase 1	90	66.6	66.6
	Phase 2	90	76.6	46.7

^*^Dark green: cases in which the increase in the number of files for training led to an increase in predictive value.

^Δ^Red frame: cases in which the predictive value exceeded the expected parameter for humans.

^†^Light green: cases in which an equality of predictive value between Phase 1 and Phase 2 was found.

First of all, it is possible to observe that, leaving aside the field of healthy skin introduced as a “control,” in 50% of cases correct answer rate higher than 88% was achieved.

In any case, it is worth considering that some of the categories investigated are new research topics (single vs. multiple charge ammunition GSW), and that the introduction of the “healthy skin” variant also represents an unprecedent variable. Surprisingly the lowest statistical values (and, consequently, the highest wrong answer rate) were obtained by this category.

With constant reference to the parameters illustrated in Table 18, it is worth emphasizing that the extension of the software training process led to an increase in the accuracy rate in 62.5% of cases related to shooting categories, that in 12.5% of cases there was a substantial invariance, and that only in 25% of cases there was a worsening of the starting value.

This makes even more evident an undoubted limitation related to this investigation, namely the scarcity of images at our disposal. To overcome this limitation, future work will focus on expanding the dataset through collaboration with other forensic institutions. It will be vital to incorporate a wider range of wound types and anatomical regions, and to develope a pre-trained model capable of distinguishing physiological skin features from pathological ones. Regarding the risk of overfitting, we acknowledge that the limited dataset and the use of data augmentation may predispose the model to memorization rather than generalization. Future iterations will benefit from benchmarking against alternative architectures and consistent cross-validation strategies.

Comparing the artificially extended database available to the Institute (240 images) with the thousands included in the studies already cited (9, 19), it is logical to expect less significant results, burdened by the greater error rate related to the small sample at our disposal.

This phenomenon, moreover, is further amplified by the “physiological imbalance” related to certain injury categories compared to others (22).

Well, observing what was obtained at the end of the study of Field 1. Regarding the “GSW” category, the highest correct answer rate of the study was recorded, equal to 96% at the end of the first session (limited training) and 100% at the end of the second session (extended training).

This result is further enhanced by the observation of an increasing trend of correct answers during the first session (Table 2), and stable during the second session because free of cognitive errors by the algorithm (Table 3). It is necessary to specify that this result is among the highest reported in preliminary studies, although further validation on larger datasets is necessary.

By interpolating the overall data relating to both categories, a surprising correct answer rate of 91.7% was achieved (Table 10).

However, the gap between the percentages of correct answers obtained by the “GSW” category compared to the “healthy skin” category remains evident (Figure 2).

In particular, regarding the second, a decreasing trend was observed in the percentage of correct answers between the first and second sessions. This unexpected result requires a weighted interpretation: the presence of skin folds, appendages (hairs, nipples) and nevi represented a confounding parameter for the algorithm. As a cause, a non-negligible number of incorrect answers obtained from the “shooting” categories derived from the erroneous interpretation as “healthy skin.”

Secondly, it is possible to note that a decrease in the rate of correct answers during the advancement of the test phase was highlighted only during the first study session of Field 1. In this case, a small number of training samples may have been insufficient for the algorithm to identify the salient parameters of human skin.

Moreover, excluding the control skin category would limit the model’s ability to distinguish pathological from non-pathological features, which is essential for our main goal (forensic reliability). For this reason, the ability of the “machine” to identify a physiological pattern could not be considered negligible.

Moreover, the integration of AI into forensic workflows could help reduce inter-observer variability, especially in complex or borderline cases. By providing consistent pattern recognition, AI tools may enhance the reliability of forensic conclusions and support their admissibility in court.

Therefore, despite the results related to the low degree of cognitive accuracy of the healthy epidermal surface, it is good to consider that this represents an important starting point for future studies of refinement.

In the future, developing a larger pool of images will certainly optimize machine training phase.

Furthermore, future development should aim to integrate image-based classification with other forensic data sources, such as radiological imaging, histopathological findings, and ballistic reports. This multi-modal fusion could enhance the forensic validity and practical utility of AI models in casework.

Regarding the results emerging from the analysis of the data relating to Field 2, the observations that can be made are multiple. Regarding the first test session, following the limited administration of 15 images for training, a decrease in the rate of positive answers was observed for the “entry GSWs” category during the advancement (Figure 3). Similarly to what was stated regarding Field 1, this is justifiable based on the limited training provided to the algorithm.

Not surprisingly, in the subsequent session with extended training (45 images) an increase in the rate of correct answers was recorded for all three categories involved (“entry GSW,” “exit GSW” “healthy skin”) as shown in Figure 3.

However, an increase in the percentage difference between the correct answers related to the “wounds” categories was recorded, equal to 10.1% (3.3 vs. 13.4). Even in this circumstance it is possible to justify the data in light of the limited database made available for training, i.e., not sufficiently representing that variability of morphological elements useful for a fine distinction between entry and exit GSWs.

Moreover, the statistical interpolation obtained from data analysis (Tables 11, 12) also showed the effectiveness exerted by the extension of the training data on final results. For both categories investigated, in fact, Phase 2 revealed a significant increase compared to Phase 1 of all the parameters investigated. Specifically, accuracy underwent an increase of 7% for “entry GSWs” category, and 12.6% for “exit GSWs” one; specularly, the F1-score also underwent an increase of 0.06 and 0.12 points, respectively. Even more notable and significant was the increase in specificity values, equal to 12.1 and 17.8%, respectively.

In interpretive terms, similar values assume absolute importance because, having reached the threshold of 75 and 80%, respectively, they are close to the scientific standard reported in the literature, namely 88% reached by the teams of Queiroz Nogueira Lira and Cheng (9, 19).

Proceeding with the evaluation of the results emerging from the analysis of Field 3, it is impossible to omit how the reconstruction of the shooting distance represents an extremely delicate and arduous task even for humans, whatever their experience and preparation.

Therefore, the predictive correctness parameters reported in Figure 4 appear even more valuable than the previous ones. Both test sessions, and in particular the second, have in fact shown a high degree of correctness for all the categories investigated, including the one related to healthy skin.

A careful eye will not miss the shortcoming related to the 66.7% rate of positive answers obtained by the “intermediate shooting distance” category at the end of the second working session, which counters observations from other categories. However, it must be noted that the result depended by a final increase of uncertain answers which affected also the “long shooting distance” category, probably due to an emerging overfitting phenomenon.

However, it is good to consider the very high degree of interpretive uncertainty attributable to visual data alone when approaching such a reconstruction (especially in cases of intermediate shooting distance). It is worth considering that in this area forensic practice recommends the execution of complementary tests on a physical sample to resolve the salient aspects of the lesion.

In any case, even regarding this field, very promising data emerged from the statistical interpolation of data (Tables 13–15). In fact, with reference to the “short distance” category, it is possible to observe that the accuracy and recall parameters have exceeded the current scientific reference standard (94.1% and 94.4% as reported in Table 13) (9), that the F1-score is equal (91.7%), and that the remaining parameters are only slightly lower (precision 89.4%, specificity 88.2%).

Even from the data relating to the “long” shooting distance category, it is possible to draw the same conclusions, with accuracy and recall values (91.1% and 94.1% as reported in Table 15) only slightly lower than the standards reported by Queiroz et al. (19).

Therefore, the final comment regarding the experiment on Field 3 concerns the need to resort to chemical–physical tests on a specific sample after a preliminary AI opinion.

Well, Field 4 presented highly encouraging results. In fact, as can be seen from the data reported in Figure 5, a final rate of correct answers equal to 90% was recorded for the “single charge ammunition” category, although accompanied by a less positive rate of 76.6% for the “multiple charge ammunition” category (Table 9). This value has in any case shown a significant increase (10%, see Figure 5) between sessions, highlighting the importance of training phase.

Once more, it emerged that the characteristics of healthy skin have represented a confounding.

Tables 16, 17 show how these elements affected statistical results in terms of very low profiles from every index, especially precision (67.5% for “single charge ammunition” category and 63.8% from “multiple charge ammunition” category) and specificity (51.8% for both categories). We consider this trend a positive factor, as it testifies to the achievement of a desired problem: the challenge to the algorithm to distinguish between pathological and non-pathological elements of similar appearance.

Therefore, decline in machine performance at this point does not constitute a pitfall, but rather a demonstration of the need for a larger dataset.

## 5 Conclusion

The results obtained are highly promising and, despite the relatively small sample of data examined compared to the experiences reported in the scientific literature, have shown statistical rates comparable to those from studies with larger case histories.

It is reiterated in any case the preliminary nature of this scientific experience, to be considered as a basis for the construction of an algorithm specifically designed for this purpose, to be made available to the scientific community as an “open source” license.

While the current model is not yet suitable for courtroom use, its performance in this pilot phase suggests strong potential for future development, especially with larger and more heterogeneous datasets.

In this way, through the implementation of a larger database and the introduction of a multicenter approach, a clear improvement of all the statistical parameters investigated therein by the ResNet V2 software is hypothesized.

In addition to the need to train the AI more thoroughly and to submit to it a greater number of cases and variables on which to “reason,” the need to educate the algorithm to recognize characteristics from the universe outside forensic pathology has emerged.

Obviously, to make such an objective achievable, it is vital to proceed with the development of a specific software pre-trained on the basis of natural elements and secondarily directed to the recognition of the typical characteristics of GSWs.

The possibility, for future research, of extending the implementation procedure illustrated in this study to further and different areas related to the forensic sphere should not be overlooked, as several alternative pattern recognition employments yet exist on the field (e.g., odontology, anthropology, diatoms taxonomy) (5–7).

In any case, the currently desirable use for the software in question would still be limited to the educational field and scientific research, but it is believed that these represent the strengths of the illustrated project as they are suitable for the definitive improvement of the system.

A key objective for future development will be the implementation of benchmarking protocols to identify the most suitable model architecture for forensic wound classification, balancing accuracy, speed, and explainability. We also recognize the need for multi-modal AI systems that incorporate contextual forensic variables.

Our current model represents a foundational step toward such integration, with future iterations aimed at combining visual data with ballistic and anatomical inputs. In future iterations, we aim to develop a decision-support interface that allows forensic experts to interact with AI outputs, validate predictions, and incorporate them into case reports. This would represent a meaningful step toward actionable support in criminal investigations and judicial proceedings, especially when images are the only available source of evidence (e.g., recorded tapes, forensic photos from investigations ended with corpses disposal).

On the other hand, a prodromal use of similar technologies could be useful in terms of automatic recognition of photographic patterns, AI-guided storage and rapid recovery of homogeneous files in case of need for comparison by the professional.

To further validate our approach, we plan to benchmark DL performance against traditional image analysis methods and incorporate interpretability tools to support forensic decision-making.

As a matter of fact, the technological progress currently achieved does not yet possess sufficient predictive capacities to allow its use in the judicial field, although the diagnostic-therapeutic sphere of the medical universe already makes extensive use of it. However, the continuous, rapid, and ever faster improvement of the computer universe bodes well for a predictable arrival of artificial intelligence in the courtrooms, at least in certain conditions characterized by lack of material sources as direct evidence.

In any case, this pilot study consolidates several classification objectives previously addressed in separate works, offering a foundation for future integrated forensic AI models, has considered exclusively the human subject, has employed the best cognitive software currently available, and has laid the foundations for the realization of a multicenter collaborative project by preparation of a reproducible protocol and, at the earliest opportunity, the interinstitutional sharing of photographic databases for the enlargement of present study cohort, starting from our yet existing working net.

## Data Availability

The data analyzed in this study is subject to the following licenses/restrictions: judicial restriction for forensic real cases. Requests to access these datasets should be directed to francesco.sessa@unict.it.
